# Identification and comparative analysis of complement C3-associated microRNAs in immune response of *Apostichopus japonicus* by high-throughput sequencing

**DOI:** 10.1038/srep17763

**Published:** 2015-12-04

**Authors:** Lei Zhong, Feng Zhang, Yu Zhai, Yanhui Cao, Si Zhang, Yaqing Chang

**Affiliations:** 1 Key Laboratory of Mariculture & Stock Enhancement in North China’s Sea, Ministry of Agriculture, Dalian Ocean University, Dalian, 116023, P.R. China

## Abstract

MicroRNAs (miRNAs) are important effectors in mediating host–pathogen interaction. In this report, coelomocytes miRNA libraries of three Japanese sea cucumbers *Apostichopus japonicus* were built by Illumina^®^ Hiseq2000 from different time points after lipopolysaccharide challenge (at time 0 h, 6 h and 12 h). The clean data received from high throughput sequencing were used to sequences analysis. Referenced to the *Strongylocentrotus purpuratus* genome, 38 conserved miRNAs were found, and three miRNA candidates were predicted by software. According to the evidence resulting from the expression of AjC3, expressing levels of spu-miR-133, spu-miR-137 and spu-miR-2004 altered along with the expression of AjC3 changing at different time points after LPS injection. Thus, we speculated that the three miRNAs may have influence on *A. japonicus* complement C3. The spu-miR-137 and miR-137 gene family in miRBase were analyzed by bioinformatics. There is an obvious discrepancy between invertebrates and vertebrates. The first and ninth nucleotides in invertebrate miR-137 are offset compared vertebrate miR-137. Importantly, this is the first attempt to map the stage of immune response regulome in echinoderms, which might be considered as information for elucidating the intrinsic mechanism underlying the immune system in this species.

Given the phylogenetic position (invertebrate deuterostomes) of the sea urchin *Strongylocentrotus purpuratus*, it is used as a model organism for developmental and evolutionary studies. However, the Japanese sea cucumber *Apostichopus japonicus,* which also belongs to the Echinodermata, has not received the same amount of attention. Thus, there are few reports available about the immune system of *A. japonicus.*

MicroRNAs (miRNAs) are a class of small (~22 nt) non-coding RNA molecules involved in the regulation of gene expression at the post-transcriptional level by degradation or translational repression^1^. miRNAs typically target 3′ untranslated regions (UTRs) of genes encoding proteins and usually downregulate their expression according to protein levels either by inhibiting mRNA translation or by increasing their own degradation rate^2^. Since the first miRNA was discovered in *Caenorhabditis elegans*^3^, a huge amount of miRNA entries has been uploaded to the miRBase (http://www. mirbase.org). Many studies indicated that miRNAs had important roles in a wide range of biological processes, including embryonic development, cell differentiation, apoptosis^4^ and immune response^5^. Although evolution was accompanied by the loss and gain of specific miRNAs^6^, some orthologous miRNAs in different species have similar or even the same sequences, which leads to the conservation of function of these miRNAs^7^. Therefore, discovery of miRNA provides a new starting point to investigate the process of immune response in sea cucumber and the evolution of the immune system.

We present the sequences of miRNAs from the coelomocytes *A. japonicus* and analyze some related to the immune response. Our results increase the available information on echinoderm immunity and provide new information for the comparative and evolutionary aspects of immunity.

## Results and Discussion

### Length distribution of miRNA sequencing libraries

**Figure 1:**
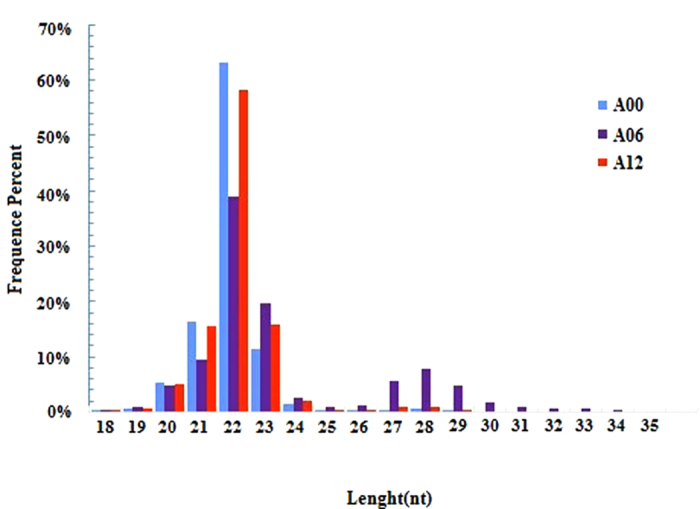
Size distribution of sequencing reads in three libraries. Different colors represent different libraries.

We built and sequenced three libraries from sea cucumber small RNAs: A00, A06 for injecting lipopolysaccharide (LPS) after 6 h and A12 for injecting LPS after 12 h. High-throughput sequencing yielded 16,385,967 clean reads (after elimination of adaptor and low-quality reads) from A00, 14,436,519 from A06 and 16,360,110 from A12. The sequence length distribution in the three libraries showed a wide variation ranging from 18 to 35 nt, in which small RNAs of 22 nt were enriched followed by 21 nt and 23 nt in all libraries (Fig. 1). Our results were consistent with the typical size of miRNAs in other reports; 22 nt miRNAs were predominant in small RNA libraries of *Blattella germanica*^8^, *Locusta migratoria*^9^, *Aedes albopictus* and *Culex quinquefasciatus*^10^ and there was a peak at 28 nt in three libraries (A00, A06 and A12). This type of small RNA (28 nt long RNAs) falls into small scan RNA (scnRNA)^11^ and *Tetrahymena* had an abundance of 28 nt long RNAs, implying the longer RNAs might be excluded from RNA-induced silencing complex (RISC) and thus available to guide other complexes that target nucleic acids in a nucleotide sequence-specific manner^12^.

### Diverse miRNA of the sea cucumber

To identify the miRNA annotation of *A. japonicus*, the genome of *S. purpuratus* was used as a reference for miRNA mapping because both *S. purpuratus* and *A. japonicus* belong to the Echinodermata and *S. purpuratus*, whose genome has been identified, is the closest species to *A. japonicus* but no sequenced genome of *A. japonicus* is available. In all, 38 known miRNAs were identified from all libraries (Table 1). Among these conserved miRNAs, in all three libraries the proportions of some miRNAs, such as spu-let-7, spu-miR-2004, spu-miR-2012 and spu-miR-71, were far greater than others. Most of these miRNAs experienced fluctuation in three time points; for instance, the figure for spu-miR-184 jumped from ~4.5% (A00) to nearly 25% (A06) and then fell considerably to ~5.8% (A12). However, the tendency of change in spu-miR-2012 was opposite to that of spu-miR-184, which reached its lowest point of 4.3%, in A06. The other miRNAs showed increasing or decreasing trends of change. These changes suggest miRNAs participate in the regulation of physiological processes. In addition, given the fluctuation of spu-let-7 between 16.1% and 12.5%, and recent studies showed let-7 miRNA was involved in cholangiocyte immune responses against *Cryptosporidium parvum* infection and white spot syndrome virus (WSSV) challenge^13^, we speculate that spu-let-7 is likely to take part in regulation of the complement system in *A. japonicus.*

The read counts showed the ratios of spu-let-7 to spu-mir-125-5p across all three libraries were nearly identical; further, these miRNAs had similar trends of changed read counts. Thus, we suggest expression of let-7 and mir-125 in *A. japonicus* are the same as in *D*. *melanogaster*^14^.

**Figure 2:**
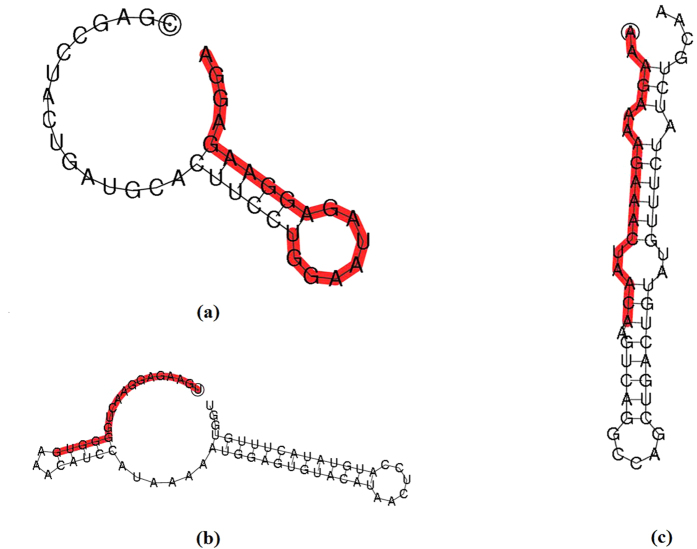
The predicted hairpin structures. The figures illustrate the predicted hairpin structures of novel-36 (**a**), novel-44 (**b**) and novel-45 (**c**), and the red parts reflect mature sequences.

After filtering the low-quality adaptor sequences of <15 nt and identifying conserved miRNAs, the matched sequences were analyzed to predict novel miRNAs using miREvo and mirdeep2 software (Novogene, Beijing, China), which identified three novel miRNA candidates. The secondary structures of these novel candidates are shown in Fig. 2. These novel miRNA candidates should be validated further by direct cloning to circumvent the inability of bioinformatics analysis to predict the sequence of mature miRNA precisely.

In all, 41 miRNAs were identified or predicted, which includes 38 known miRNAs and three novel miRNAs. The sea cucumber miRNAs had a small distribution of length centered on 22 nt (Table 1). With the exception of two miRNA pairs (spu-miR-29a,b and spu-miR-92a,c; and spu-miR-29a,b and spu-miR-92b-3p, the miRNAs had unique sequences at nucleotides 2–8, suggesting marked diversity of miRNA targeting in this simple animal. From the pool of *A. japonicus* miRNAs, we found some, including spu-miR-2004 and spu-miR-2005, were present only in Echinodermata according to the miRBase records. Similar phenomena in Drosophila and Euarchonta could be explained if transposition and retrotransposition affect the gain and loss of miRNAs^15^,^16^. According to the results of this study, we speculate one of the mechanisms of miRNA’s molecular evolution has been formed since the emergence of the Echinodermata or earlier.

Compared to the results of earlier work with *A. japonicus* miRNA^13^,^17^, the varieties of both conserved and novel miRNAs in this report are fewer. These differences could be due to two main reasons: discrepant samples and developmental stage can influence the results of separate experiments. Healthy adults’ coelomocytes was the material from which the total RNA was isolated in this work, whereas ulcerated skin and respiratory tree were used in the two earlier studies. Further, limited genome information, different software and different parameter settings during data processing can all influence the results.

### Phylogenetic analysis of the miR-137 family

No miRNA in this study targeted *S. purpuratus* complement C3, which was expected. Although *S. purpuratus* and *A. japonicus* belong to the Echinodermata and *S. purpuratusis* is the closest species to *A. japonicus*, the similarity of the two animals’ C3 gene is very low (7%). Because miRNAs pairing to recognition of targets must comply with the sequence in recognition^18^, any change in the area of recognition will alter miRNAs that regulate the target gene.

**Figure 3:**
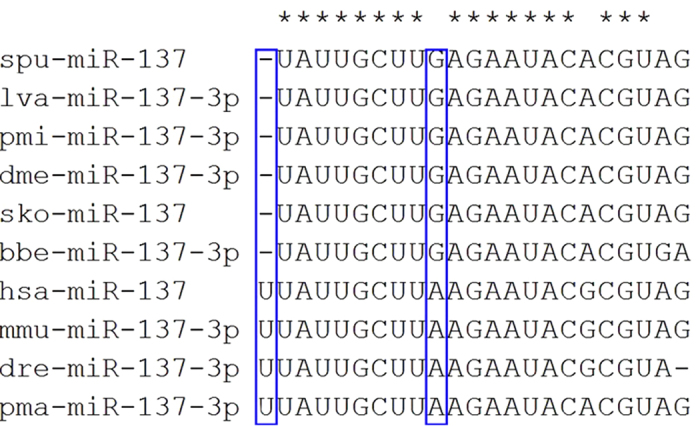
Alignment of the mature miR-137. Asterisks (*) above the alignments indicate the positions of identity. Frames represent the different bases in vertebrate miR-137 at the first and ninth positions.

**Figure 4:**
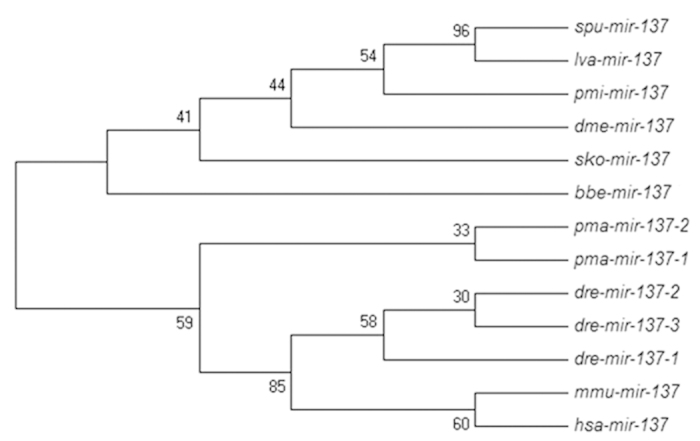
Phylogenetic relationships among members of the full-length sequences of miR-137 family hairpin. The tree was constructed using the Maximum Parsimony program from an alignment done with CLUSTAL W. Accession numbers for the sequence used in this alignment are given in Table 2.

According to the results of this study, the sequences of mature miR-137 and miR-137 hairpin in *A. japonicus* are the same as those in *S. purpuratusis*; therefore, mature and hairpin sequences of spu-miR-137 were used to represent the sea cucumber in Fig. 3 and 4. The IDs of sequence of mature and hairpin miR-137 in miRBase are shown in Table 2.

As exemplified by spu-miR-137, the first nucleotide was offset compared to vertebrate members of the miR-137 family (Fig. 3). Further, the first to eighth bases of invertebrate nucleotides are the same as nucleotides 2–9 in vertebrates. Because miRNA targeting is defined primarily by nucleotides 2–8^19^, the absence of base 1 in vertebrates is thought to alter target recognition substantially less.

In miRBase, 95 mature sequences of miR-137 occur in 57 species, which is a highly conserved miRNA. To identify the evolutionary relationship within the miR-137 family, the full-length sequences of spu-miR-137 hairpins need to be aligned with miR-137 of other species. We undertook phylogenetic analysis. Before creating the cladogram of miR-137, we collected miR-137 of several model organisms. Evidence resulting from Davide’s study^20^ verified that Maximum Parsimony is suitable for the analysis of this data set. Thus, comparisons within the miR-137 group were subject to Maximum Parsimony analysis combined with a Bootstrap framework (Fig. 4). The cladogram showed there were two main branches. One branch consisted of only invertebrate miR-137 and all vertebrate miR-137s constituted the other branch. In the invertebrate branch, miR-137s of echinoderms, including the sea cucumber, were in the same clade and shared a relatively close ancestral linkage with *D. melanogaster*, which is less developed than the Echinodermata. This result implies miR-137s of Echinodermata and Arthropoda share a common ancestor. Combined with Fig. 3, we suggest the first base lacking in lower animals’ miR-137 and the ninth base in invertebrate miR-137 changing (from G to A), the tenth base in vertebrate miR-137, are probably the main reason why the result arises in the cladogram. Doench *et al.*^21^ suggested miRNAs combining with target sequences will benefit from the non-conservative bases located in nucleotides 9–11. Discrimination between these two alternatives needs further study.

### Differential expression of miRNAs among three libraries

By analyzing known miRNAs and novel miRNAs from this experiment using bioinformatics, we predicted the target genes of five miRNAs were *A. japonicus* complement C3 or *A. japonicus* complement C3–2; they are novel-45, spu-miR-133, spu-miR-137, spu-miR-2004 and spu-miR-2006.

**Figure 5:**
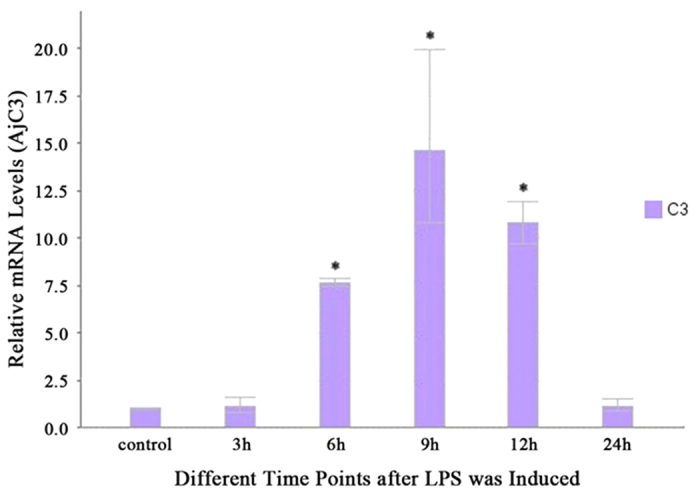
Coelomocyte AjC3 gene expression at different times after LPS challenge. Each vertical bar represents the mean ± S.D. (*n* = 3) for various time points. Statistically significant differences in different groups are indicated by an asterisk (*) at *P* < 0.05.

C3 is known as functional protein of the complement system. To understand the expression of AjC3 in sea cucumber coelomocytes, we detected the expression of AjC3 among different time points after inducing LPS in coelomocytes by RT–PCR. In this study, the sequences of RT–PCR primer were show in Table 3. As shown in Fig. 5, in the first 3 h, the expression of AjC3 was almost the same compared to the control, indicating the complement system in *A. japonicus* was not activated. However, at the second time point (6 h), the expression was five times as high as the former level. We believe an immune response associated with AjC3 occurred in this period in response to the lipopolysaccharide (LPS) challenge after 3–6 h. The level of expression was maximal at the third time point (9 h). Correspondingly, the immune response became more pronounced. At the remaining time points, the levels of expression began to fall. The tendency was in accord with the study^22^ in which LPS induced an immune response in *S. purpuratusis.* This result indicates that AjC3 participated in the immune response.

**Figure 6:**
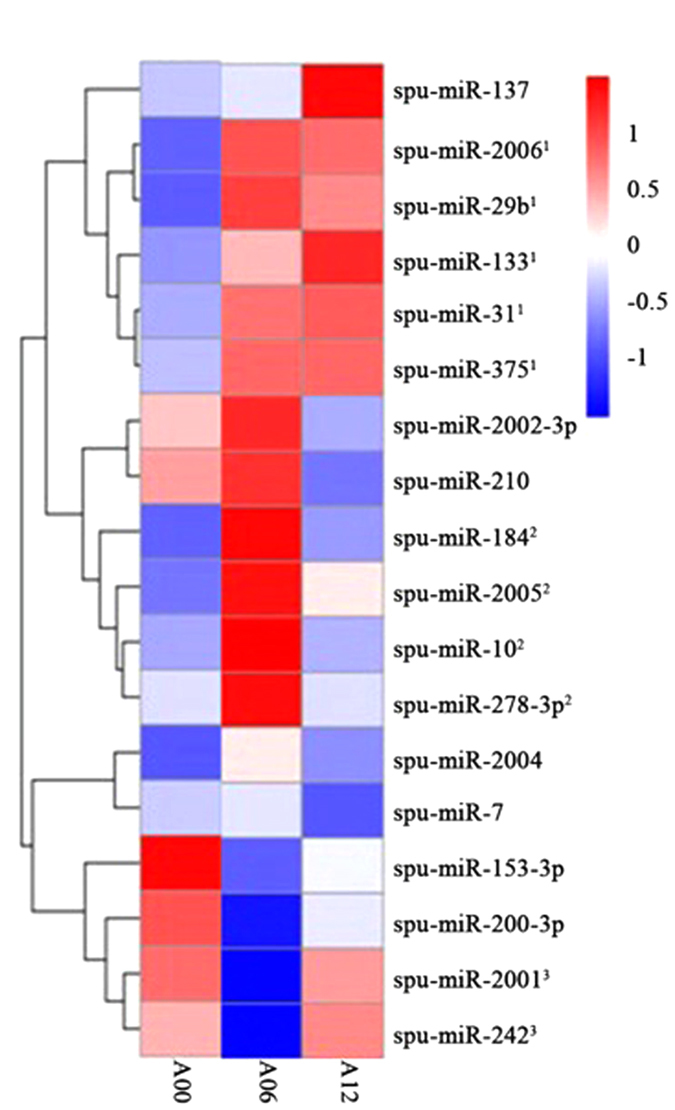
Heat map of miRNAs related to the immune response. Heat map showing the differential expression of miRNAs in three libraries (log_2_ transformed relative expression values). miRNA with a higher expression level is mapped to the red part and miRNA with a lower expression level is mapped to the blue part.

On the basis of the evidence resulting from the expression of AjC3, we selected time points 6 h and 12 h to mark the gene’s (AjC3) increasing and declining process in the immune response, respectively, which helped us compose libraries to detect differential expression of miRNAs when the sea cucumber was infected by bacteria. The results are given in Table 4 and illustrated by Fig. 6. Three libraries (A00, A06 and A12) were pairwise aligned by the DEGseq (2010) R package (R Development Core Team, 2010) for which the threshold was described in Materials and Methods. Table 4 gives the miRNAs expressed differentially in three alignments. As no miRNA was down-regulated in A12 vs. A00, the result is not given in Table 4. spu-miR-133 and spu-miR-137 were overexpressed in A12 vs. A00 (Table 4) and the expression of AjC3 showed a tendency to decrease at the fourth time point, 12 h (Fig. 5). Moreover, at the second time point (6 h), two down-regulated miRNAs, spu-miR-133 and spu-miR-137, were calculated in A06 vs. A12; however, the level of AjC3 began to increase. On the basis of these results, we believe the two miRNAs likely influence the expression of AjC3, which is predicted by bioinformatics to be the target gene of spu-miR-133 and spu-miR-137. It is noteworthy that the trend of miR-2004 in Table 1 is negatively correlated with the change of level of AjC3 (Fig. 5), but there is no miR-2004 in Table 4. We suspect spu-miR-2004 takes part in the regulation of the expression of AjC3 as well. Although spu-miR-133 is one of the up-regulated miRNAs in A06 vs. A00, given the read counts (adjusted by trimmed mean of M-values (TMM) in both columns, A06 and A00, miR-133 could not affect the expression of AjC3 very much, when LPS was challenged after 6 h.

In addition, we have cloned *A. japonicus’* factor B and proved it involves an immune response^23^, so we believe there is an alternative pathway complement in the sea cucumber. C3 can be compounded slightly in an organism; factors B and D can interact to produce C3 convertase, but the activity of C3 convertase can be suppressed by factors H and I. This mechanism maintains the amount of C3 in dynamic equilibrium in a healthy organism. The analytical results showed that spu-miR-133 and spu-miR-2006 were up-regulated in the sea cucumber at 6 h and 12 h after challenge with LPS. Most miRNAs have more than one target mRNA and we suggest these two miRNAs’ target mRNA might be factor H and/or factor I. Thus, the two factors were suppressed at 6 h, which improved the level of C3 and had an effect on AjC3 mRNA at 12 h. This possibility needs to be examined by further experiments.

Figure 6 shows a heat map corresponding to the relative abundance of overlapping miRNAs among the A00, A06 and A12 libraries. The miRNAs can be classified into three main groups on the basis of their expression trends; (1) relatively high abundance in both A06 and A12, (2) relatively high abundance in only A06, and (3) relatively high abundance in both A00 and A12. Overall, we found the first and second groups had almost the same number of miRNAs, which occupy the majority of miRNAs in the heat map. This indicates these miRNAs might take part in the immune response of *A. japonicus*. The main function of the third group of miRNAs is to maintain some genes in normal amounts.

Out of the six miRNAs highly expressed in all three libraries, nine are relatively highly expressed in at least two of the three libraries, which may indicate overlap in the post-transcriptional gene regulatory mechanisms. We note this is the first attempt to map the stage of immune response related to complement C3 in echinoderms, and temporal expression might vary even among the miRNAs that appear to be abundant in all stages.

## Materials and Methods

### Animals and LPS treatments

Adult sea cucumbers (10 cm in length) were collected from the coastal waters of Heishijiao (Dalian, China) in late November and were kept in seawater aquaria at 16 °C–19 °C. The sea cucumbers were injected once with 0.5 ml LPS solution (Sigma, Sangon Biotech, Shanghai, China), which was prepared by dissolving 10 mg LPS in 5 ml sterile seawater. Since the average volume of coelomic fluid inside a 10 cm long sea cucumber is 7 ml^24^, the LPS concentration can be estimated to be 0.13 mg/ml of coelomic fluid. The control group was injected with 0.5 ml sterile seawater.

Coelomocytes were collected at 3, 6, 9, 12 and 24 h after injection according to the methods reported by Francisco Ramírez-Gómez *et al.* (2008), with some adjustments, as follows: the coelomocytes were collected over ice by making an incision on the anterior (tentacled) end of the animals and decanting the coelomic fluid on a clean culture dish. The coelomic fluid was collected in sterile 1.5 ml centrifuge tubes and centrifuged at 3780 × *g* for 3 min at 4 °C. The pelleted coelomocytes were kept in liquid nitrogen for RNA extraction.

### RNA isolation, library preparation and sequencing

#### RNA isolation

Total RNA was isolated using the RNAiso Plus kit (Takara, Dalian, China) according to the methods reported by Lifeng Ji *et al.* (2009)^25^.

#### RNA quantification and qualification

Novogne (Beijing, China) performed the RNA extraction, library preparation, and sequencing analyses. Firstly, RNA degradation and contamination was monitored on 1% agarose gels. Then, RNA purity was checked using the NanoPhotometer^®^ spectrophotometer (IMPLEN, CA, USA). Next, the RNA concentration was measured using the Qubit^®^ RNA Assay Kit in the Qubit^®^ 2.0 Flurometer (Life Technologies, CA, USA). Finally, RNA integrity was assessed using the RNA Nano 6000 Assay Kit with the Agilent Bioanalyzer 2100 system (Agilent Technologies, CA, USA).

#### Library preparation for Small RNA sequencing

Three micrograms of total RNA per sample were used as input material for the creation of small RNA libraries. Sequencing libraries were generated using a NEBNext^®^ Multiplex Small RNA Library Prep Set for Illumina^®^ (New England Biolabs (NEB), MA, USA) following the manufacturer’s recommendations. Index codes were added to identify the sequences from each sample. Briefly, NEB 3′ SR adaptors were directly and specifically ligated to the 3′ end of all miRNA, siRNA and piRNA. After the 3′ ligation reaction, the SR RT primer hybridized to the excess 3′ SR adaptor that remained free after the 3′ ligation reaction and transformed the single-stranded DNA adaptor into a double-stranded DNA molecule. This step is important to prevent adaptor-dimer formation. Additionally, dsDNAs are not substrates for ligation mediated by T4 RNA Ligase 1 and therefore do not join with the 5′ SR adaptor in the subsequent ligation step. The 5′-end adapter was ligated to 5′ ends of all miRNA, siRNA and piRNA. Then, first-strand cDNA was synthesized using M-MuLV Reverse Transcriptase (RNase H-). PCR amplification was performed using LongAmp Taq 2× Master Mix, SR Primer for Illumina and index (X) primer. PCR products were purified on an 8% polyacrylamide gel (100 V for 80 min). DNA fragments corresponding to approximately 140–160 bp (the length of small noncoding RNA plus the 3′ and 5′ adaptors) were recovered and dissolved in 8 μL elution buffer. Finally, library quality was assessed on the Agilent Bioanalyzer 2100 system using DNA High Sensitivity Chips.

#### Clustering and sequencing

Clustering of the index-coded samples was performed on a cBot Cluster Generation System using TruSeq SR Cluster Kit v3-cBot-HS (Illumia) according to the manufacturer’s instructions. After cluster generation, the library preparations were sequenced on the Illumina Hiseq 2500/2000 platform and 50 bp single-end reads were generated.

#### Quantitative real-time PCR

The sequence of *AjC3* was cloned and uploaded to NCBI (GenBank: HQ214156.1). Based on its sequence, we designed primers for RT–PCR.

To compare mRNA levels between normal culture conditions and the LPS treatment, equal amounts of cDNA template were used in quantitative real time PCR (qRT –PCR). *Cytochrome b* (*Cytb*) was used as the reference gene^26^, and *AjC3* was studied using qRT–PCR with the Applied Biosystems Step One Real-time PCR System (Applied Biosystems, USA). The reactions were performed in a total volume of 20 μl, containing 10 μl of 2× SYBR Green Master mix (SYBR PrimeScript^TM^ RT–PCR Kit II, TaKaRa) and 0.4 μl of Rox Reference Dye (50×, 6 μl dH_2_O, 2 μl cDNA template and 0.8 μl of each primer). The qRT–PCR program was 95 °C for 30 s, followed by 40 cycles of 95 °C for 5 s and 60 °C for 30 s. Melting curve analysis of the amplification products was performed at the end of each PCR reaction to confirm that only one PCR product was amplified and detected. The relative mRNA levels of the target genes were calculated as 2^−ΔΔCt ^27. Statistical calculations were performed using SPSS (version 17.0). A significant difference was indicated by one-way ANOVA (*P* < 0.05).

### Data analysis (Novogene Gene Regulation Department)

#### Quality control

Raw data (raw reads) in fastq format were first processed using custom perl and python scripts. In this step, clean data (clean reads) were obtained by removing reads containing poly-N tails or 5′ adapter contaminants, reads without 3′ adapters or insert tags, reads containing poly-A, T, G, or C, or low quality reads from the raw data. At the same time, the Q20, Q30, and GC-content of the raw data were calculated. Then, we chose a certain range of lengths from the clean reads on which to do all the downstream analyses.

#### Reads mapping to the reference sequence

The small RNA tags were mapped to the reference sequence by Bowtie^28^ without mismatches to analyze their expression and distribution on the reference.

#### Known miRNA alignment

Mapped small RNA tags were used to look for known miRNAs. miRBase20.0 was used as reference. The modified software mirdeep2^29^ and srna-tools-cli were used to obtain potential miRNAs and draw secondary structures. Custom scripts were used to obtain miRNA counts. Since the position of the first base in miRNA hairpin structures depends on the length of the miRNA, we also determined the base bias on the first position of the identified miRNA of certain lengths and on each position of all identified miRNA, respectively.

#### Remove tags from these sources

To remove tags originating from protein-coding genes, repeat sequences, rRNA, tRNA, snRNA, and snoRNA, small RNA tags were mapped to RepeatMasker, the Rfam database or those types of data from the specified species itself.

#### Novel miRNA prediction

The characteristics of hairpin structures of miRNA precursors can be used to predict novel miRNAs. The available software, miREvo^30^ and mirdeep2^29^, were integrated to predict novel miRNA through exploring secondary structures, Dicer cleavage sites and minimum free energy of small RNA tags unannotated in the former steps. At the same time, custom scripts were used to obtain counts of the identified miRNA as well as base bias on the first position with certain lengths and on each position of all identified miRNA, respectively.

#### Small RNA annotation summary

We summarized all alignments and annotations that were previously obtained. In the alignment and annotation steps, some small RNA tags may be mapped to more than one category. To make sure that unique small RNAs mapped to only one annotation, we followed the following priority rule: known miRNA > rRNA > tRNA > snRNA > snoRNA > repeat > gene > NAT -siRNA > gene > novel miRNA > ta-siRNA. The total proportion of rRNA was used as a marker of sample quality. In general, high quality is indicated by less than 60% rRNA in plant samples and less than 40% in animal samples.

#### miRNA editing analysis

In mature miRNA, the position between approximately 2 to 8 bp is called the seed region, which is highly conserved. The target of an miRNA might be different if the nucleotides in this region vary. In our analysis pipeline, miRNAs containing potentially edited bases could be detected by aligning all the small RNA tags to mature miRNAs, and allowing one mismatch.

#### Target gene prediction

Predicting target genes of the identified miRNAs was performed by psRobot_tar in miRanda^31^ for animals.

#### Quantification of miRNA

miRNA expression levels were estimated in transcripts per million using the following formula^32^ for normalized expression:

mapped read count/total number of reads × 1000000.

#### Differential expression of miRNA

Differential expression analysis of two samples was performed using the DEGseq (2010) R package. The *P*-value was adjusted using the q-value^33^. A q-value <0.01 and |log2(foldchange)| > 1 were set as the thresholds for significantly differential expression by default.

## Additional Information

**How to cite this article**: Zhong, L. *et al.* Identification and comparative analysis of complement C3-associated microRNAs in immune response of *Apostichopus japonicus* by high-throughput sequencing. *Sci. Rep.* **5**, 17763; doi: 10.1038/srep17763 (2015).

## Figures and Tables

**Table 1:** Conserved and novel miRNAs identified from sea cucumber.

miRNA	Sequence	Read counts in A00 (%)	Read counts in A06 (%)	Read counts in A12 (%)	Length (nt)
spu-let-7	UGAGGUAGUAGGUUAUAUAGUU	15.1390%	12.5327%	16.1154%	22
spu-miR-1	UGGAAUGUAAAGAAGUAUGUAU	0.2449	0.3768	0.2438	22
spu-miR-10	AACCCUGUAGAUCCGAAUUUGUG	0.0683	0.3046	0.0715	23
spu-miR-124	UAAGGCACGCGGUGAAUGCCA	0.0001	0.0000	0.0003	21
spu-miR-125-5p	UCCCUGAGACCCUAACUUGUGA	6.7202	5.6355	6.6009	22
spu-miR-133	UUUGGUCCCCUUCAACCAGCCGU	0.0018	0.0066	0.0192	23
spu-miR-137	UAUUGCUUGAGAAUACACGUAG	0.0018	0.0021	0.0112	22
spu-miR-153-3p	UUGCAUAGUCACAAAAGUGAUU	2.6276	0.9457	1.3430	22
spu-miR-184	UGGACGGAGAACUGAUAAGGGC	4.6515	24.7072	5.7638	22
spu-miR-200-3p	UAAUACUGUCUGGUGAUGAUGUU	12.1416	6.4098	8.8607	23
spu-miR-2002-3p	UGAAUACAUCUGCUGGUUUUUAU	0.0020	0.0031	0.0012	23
spu-miR-2004	UCACACACAACCACAGGAAGUU	8.6233	11.8936	9.4464	22
spu-miR-2005	AGUCCAAUAGGGAGGGCAUUGCAG	0.0095	0.0354	0.0168	24
spu-miR-2006	GAGCACACUUGGUAGCGGUGCC	0.0000	0.0028	0.0020	22
spu-miR-2007	UAUUUCAGGCAGUAUACUGGUAA	0.0431	0.0349	0.0276	23
spu-miR-2008	AUCAGCCUCGCUGUCAAUACG	0.0003	0.0006	0.0004	21
spu-miR-2009	UGAGUUGUCCCACAAAGAACACA	0.0000	0.0000	0.0000	23
spu-miR-2010	UUACUGUUGAUGUCAGCCCCUU	0.0048	0.0069	0.0068	22
spu-miR-2011	ACCAAGGUGUGCUAGUGAUGAC	16.6887	4.3260	14.5276	22
spu-miR-2012	UAGUACUGGCAUAUGGACAUUG	8.9247	7.8812	8.2617	22
spu-miR-2013	UGCAGCAUGAUGUAGUGGUGU	0.0280	0.0180	0.0177	21
spu-miR-210	UUGUGCGUGCGACAGCGACUGA	0.2122	0.2826	0.1201	22
spu-miR-242	UUGCGUAGGCGUUGUGCACAGU	0.0013	0.0002	0.0016	22
spu-miR-252a	CUAAGUACUAGUGCCGUAGGUU	0.0007	0.0012	0.0004	22
spu-miR-252b	CUAAGUAGUAGUGCCGCAGGUA	0.0001	0.0000	0.0001	22
spu-miR-278-3p	UCGGUGGGACUUUCGUUCGAUU	0.0039	0.0796	0.0038	22
spu-miR-29a	AAGCACCAGUUGAAAUCAGAGC	0.0000	0.0001	0.0002	22
spu-miR-29b	UAGCACCAUGAGAAAGCAGUAU	0.0003	0.0031	0.0020	22
spu-miR-31	AGGCAAGAUGUUGGCAUAGCU	0.0092	0.1429	0.1914	21
spu-miR-34	CGGCAGUGUAGUUAGCUGGUUG	0.0001	0.0001	0.0000	22
spu-miR-375	UUGUUCGUUCGGCUCGCGUCAA	1.0854	5.5086	5.4916	22
spu-miR-7	UGGAAGACUAGUGAUUUUGUUGU	0.0004	0.0005	0.0001	23
spu-miR-71	UGAAAGACAUGGGUAGUGAGAUU	17.0703	11.4593	17.1849	23
spu-miR-9-5p	UCUUUGGUUAUCUAGCUGUAUG	3.0691	2.1988	2.7989	22
spu-miR-9-3p	AUAAAGCUAGGUUACCAAAGAU	0.2353	0.1982	0.1837	22
spu-miR-92a	UAUUGCACUUGUCCCGGCCUACU	0.1088	0.2126	0.1141	23
spu-miR-92b-3p	UAUUGCACUUGUCCCGGCCUGC	0.9601	1.7556	1.0126	22
spu-miR-92c	UAUUGCACUCGUCCCGGCCUGC	1.3219	3.0331	1.5563	22
Novel-36	UGGAAUAGAGGAAGAGGA				18
Novel-44	UGAAGAGGAACUGGGGUG				18
Novel-45	AAAGAAAAGAAACUAACA				18

**Table 2:** Sequences of multiple sequence alignment and constructed phylogenetic tree.

Species (abbreviation)	Mature sequence name and ID in miRBase	Hairpin sequence name and ID in miRBase
*Strongylocentrotus purpuratus* (spu)	spu-miR-137 MIMAT0009645	spu-miR-137 MI0010181
*Lytechinus variegatus* (lva)	lva-miR-137-3p MIMAT0032207	lva-miR-137 MI0025153
*Patiria miniata* (pmi)	pmi-miR-137-3p MIMAT0032160	pmi-miR-137 MI0025108
*Drosophila melanogaster* (dme)	dme-miR-137-3p MIMAT0005507	dme-miR-137 MI0005849
*Saccoglossus kowalevskii* (sko)	sko-miR-137 MIMAT0009628	sko-miR-137 MI0010165
*Branchiostoma belcheri* (bbe)	bbe-miR-137-3p MIMAT0031590	bbe-miR-137 MI0026184
*Petromyzon marinus* (pma)	pma-miR-137-3p MIMAT0019467	pma-miR-137-1 MI0017073
		pma-miR-137-2 MI0017074
*Danio rerio* (dre)	dre-miR-137-3p MIMAT0001834	dre-miR-137-1 MI0002000
		dre-miR-137-2 MI0002001
		dre-miR-137-3 MI0019582
*Mus musculus* (mmu)	mmu-miR-137-3p MIMAT0000149	mmu-miR-137 MI0000163
*Homo sapiens* (hsa)	hsa-miR-137 MIMAT0000429	hsa-miR-137 MI0000454

**Table 3:** Sequence of RT–PCR primers used in this study.

Primer	Sequence (5′ → 3′)
Cytb-F	TGAGCCGCAACAGTAATC
Cytb-R	AAGGGAAAAGGAAGTGAAAG
RT-PCR F	AATGCCAGGGAAGCGTTAG
RT-PCR R	TCCCGTTGGGCTTGTCTC

**Table 4:** Differentially expressed miRNAs in sea cucumber coelomocytes among the three libraries.

miRNA	miRNA sequencing		log_2_.fold_change.	*p* value	*q*.value.(Storey. *et al*. 2003)
Up-regulated miRNAs in A06 vs. A00					
	A06	A00			
spu-miR-10	2806.42904934567	741.820930698418	1.9196	0	0
spu-miR-184	227607.075016367	50492.8044074126	2.1724	0	0
spu-miR-375	50746.4369120932	11782.0158000191	2.1067	0	0
spu-miR-31	1316.84465932619	99.4578465553323	3.7269	4.38E-300	2.72E-300
spu-miR-278-3p	733.55228102627	41.8769880232978	4.1307	2.7239E-177	1.4663E-177
spu-miR-2005	325.957505520544	102.822961664347	1.6645	1.4215E-36	6.0414E-37
spu-miR-133	60.932165278795	19.0689856177517	1.676	4.187E-08	1.5318E-08
spu-miR-2006	25.4377000678465	0	5.6689	4.3631E-08	1.5318E-08
spu-miR-29b	28.9871465889413	3.365115109015	3.1067	2.4866E-07	8.3662E-08
Down-regulated miRNAs in A06 vs. A00					
spu-miR-153-3p	8711.52491160714	28522.7156640112	−1.7111	0	0
spu-miR-200-3p	59048.0007505138	131799.967868076	−1.1584	0	0
spu-miR-2011	39852.0023900127	181159.102385429	−2.1845	0	0
spu-miR-242	1.77472326054743	14.2082637936189	−3.0011	0.0023565	0.00073184
Up-regulated miRNAs in A06 vs. A12					
	A06	A12			
spu-miR-10	2970.02898983417	733.495990596778	2.0176	0	0
spu-miR-184	240875.361252259	59120.7570149163	2.0266	0	0
spu-miR-278-3p	776.314491440633	38.5534640937282	4.3317	1.7714E-177	1.6879E-177
spu-miR-210	2754.66432446676	1232.07722964	1.1608	6.4879E-149	4.8573E-149
spu-miR-2002-3p	30.6769436133799	12.7422466072492	1.2675	0.003471	0.0015818
Down-regulated miRNAs in A06 vs. A12					
spu-miR-2011	42175.1629277336	149013.712862067	−1.821	0	0
spu-miR-137	20.0339223597583	115.333668009204	−2.5253	7.8295E-17	4.3192E-17
spu-miR-133	64.484187595472	197.341460276372	−1.6137	1.0531E-15	5.2563E-16
spu-miR-242	1.87818022122734	16.3362135990374	−3.1207	0.00043836	0.00020885
Up-regulated miRNAs in A12 vs. A00					
	A12	A00			
spu-miR-31	1916.66168661923	91.5122564266522	4.3885	0	0
spu-miR-375	54982.3585263353	10840.7620761663	2.3425	0	0
spu-miR-133	192.622904944761	17.545582999095	3.4566	4.2218E-38	6.5052E-38
spu-miR-137	112.57596928063	17.545582999095	2.6817	2.108E-18	2.7841E-18
spu-miR-2006	20.0914619396026	0	5.3285	3.3754E-06	3.4674E-06
spu-miR-29b	19.7725498453232	3.09627935278147	2.6749	0.00024973	0.00021989
